# Comparison of transvaginal mesh surgery and robot-assisted sacrocolpopexy for pelvic organ prolapse

**DOI:** 10.1186/s12893-022-01702-z

**Published:** 2022-07-11

**Authors:** Mayuko Kusuda, Keiko Kagami, Ikumi Takahashi, Takahiro Nozaki, Ikuko Sakamoto

**Affiliations:** Department of Obstetrics and Gynecology, Yamanashi Prefectural Central Hospital, 1-1-1 Fujimi, Kofu city, Yamanashi 400-8506 Japan

**Keywords:** Laparoscopic, Pelvic organ prolapse, Robot-assisted, Stress urinary incontinence, Transvaginal

## Abstract

**Background:**

Pelvic organ prolapse (POP) is greatly affecting the quality of life (QOL) of women. There are some surgical techniques for POP repair, for example, transvaginal mesh surgery (TVM), laparoscopic sacrocolpopexy (LSC), and robot-assisted sacrocolpopexy (RSC). In the United States and Europe, the number of TVM has rapidly decreased since 2011 due to complications and safety concerns and has shifted to LSC/RSC. In Japan, RSC has increased after the insurance coverage of RSC in 2020. Therefore, we compared the surgical outcomes of TVM and RSC in POP surgery.

**Methods:**

We retrospectively collected POP surgery underwent TVM or RSC at our hospital and compared the operative time, blood loss, postoperative hospital stay, postoperative complications, and preoperative and postoperative stress urinary incontinence (SUI) of two groups. Preoperative and postoperative SUI were classified into 3 groups: “improved preoperative SUI”, “persistent preoperative SUI” and “de novo SUI”, which occurred for the first time in patients with no preoperative SUI, and compared incidence rate. The Mann–Whitney U test and Fisher’s exact test were used to compare the two groups, and P < 0.05 was considered statistically significant.

**Results:**

From August 2011 to July 2021, 76 POP surgery was performed and they were classified into two groups: TVM group (n = 39) and RSC group (n = 37). There was no difference in patient age and BMI between the TVM and RSC groups. The median of operative time was 78.0 vs. 111.0 min (p = 0.06), blood loss was 20.0 ml vs. 5.0 ml (p < 0.05), and postoperative hospital stay was 4.0 days vs. 3.0 days (p < 0.05), with less blood loss and shorter postoperative hospital stay in the RSC group. There was no difference in postoperative complications between the TVM and RSC groups (17.9% vs. 16.2%, p = 1.00). De novo SUI was 25.6% vs. 5.4% (p < 0.05) in the TVM and RSC groups, of which 23.1% vs. 5.4% (p < 0.05) occurred within 3 months of surgery.

**Conclusion:**

RSC is more beneficial and less invasive for patients with pelvic organ prolapse than TVM. In addition, de novo SUI as postoperative complication of RSC was lower than of TVM.

## Introduction

Pelvic organ prolapse (POP), which is the dropping and bulging out from the vagina of pelvic organs, causes discomfort, lower urinary tract symptoms, sexual dysfunction, defecation problems, and decreased quality of life (QOL) of patients. It has been reported that up to 50% of women over the age of 50 who have experienced multiple births have POP [1].

Surgical management of POP can be divided into non-mesh and mesh surgery. Conventional non-mesh surgeries, for example vaginal hysterectomy and colporrhaphy, were concerned high recurrence rate [2]. On the other hand, mesh surgery which includes transvaginal mesh surgery (TVM), laparoscopic sacrocolpopexy (LSC), and robot-assisted sacrocolpopexy (RSC), has been reported as a procedure with a low recurrence rate [3].

Mesh exposure and stress urinary incontinence (SUI) are reported major complications specific to mesh surgery [4, 5]. Postoperative new SUI (de novo SUI) was caused by urethral kinking and dynamic obstruction, which was a result of excessive mesh tensioning of POP surgery [6, 7].

Although TVM used to be a major technique, the U.S. Food and Drug Administration (FDA) issued warnings twice in 2008 and 2011, then banned TVM in the USA in 2014, due to complications such as damage to surrounding organs and mesh exposure caused by blind manipulation. Since then, the technique has shifted to LSC/RSC.

In Japan, TVM was introduced in 2005 and LSC and RSC were covered by insurance in 2014 and 2020, after that LSC/RSC has been increasing and becoming the main techniques. But TVM, which has been improved by mesh cutting and mesh material to overcome these complications, has been selected for suitable patients at some hospital. Faioli et al. reported that TVM with Anterior/Apical single incision mesh Elevate™ resulted in a well-tolerated procedure with a high success rate and low complications rate [8]. Also Kawaguchi et al. reported that TVM using new polytetrafluoroethylene mesh resulted in low recurrence and complication rates and more effective and safer than TVM using conventional polypropylene mesh [9].

In this study, we compared the surgical outcomes of conventional TVM and RSC and the preoperative and postoperative SUI of POP surgery.

## Subjects and methods

The patients who underwent POP surgery at Yamanashi Prefectural Central Hospital from August 2011 to July 2021 were retrospectively selected for this study according to the following inclusion criteria; (1) TVM which include Anterior TVM (A-TVM), Anterior–Posterior (AP-TVM) and A-TVM + hysterectomy, (2) RSC with total abdominal hysterectomy or supracervical hysterectomy, include patient add bilateral salpingo-oophorectomy. The exclusion criteria was; (1) Non-mesh surgery and LSC surgery, (2) underwent other surgery at the same time, (3) never see hospital after surgery. They were classified into two groups: TVM group (39 cases) and RSC group (37 cases) and compared the operative time, blood loss, postoperative hospital stay, and postoperative complications of TVM and RSC.

The severity of POP was assessed by the POP-Q (pelvic organ prolapse quantification) stage classification. The operation data, such as operative time and blood loss was counted during operation and recorded by surgeon on medical charts. Postoperative hospital stay was decided by doctors depending on condition of each patient. Postoperative complications have included mesh exposure, umbilical hernia, SUI, and recurrence of POP. In addition, we focused on SUI. We counted the presence of SUI if the doctor recorded the patient’s complaint or recorded incontinence was appeared when the patient applied abdominal pressure during examination on medical charts. SUI was classified into 3 groups according to evaluate before or after POP surgery: (1) preoperative SUI cured after POP surgery defined as “improved preoperative SUI”, (2) preoperative SUI remained after POP surgery defined as “persistent preoperative SUI”, (3) SUI occurred for the first time after POP surgery defined as “de novo SUI”. The Mann–Whitney U test and Fisher’s exact test were used to compare the two groups, and P < 0.05 was considered statistically significant. This robotic-surgery study was approved by the Ethical Committee of Yamanashi Prefectural central hospital and all patients approved informed consent that their information used for study in a form that does not identify personal information.

## Results

From August 2011 to July 2021, we had 84 POP surgery in our hospital. 76 cases were selected for this study according to the inclusion criteria, and classified into two groups: TVM group (39 cases) and RSC group (37 cases). We excluded 5 non-mesh surgery and 3 LSC from this study.

In the TVM and RSC groups, the median patient age was 72.0 vs. 73.0 years (p = 0.963) and BMI was 24.7 vs. 24.5 kg/m^2^ (p = 0.868) (Table 1). As for the POP-Q stage, stage II and stage III was the main stage in both two groups, and uterine prolapse plus cystocele was the main type in both groups, which accounted for 48.7% (19/39) in the TVM group and 35.1% (13/37) in the RSC group, and bladder prolapse alone accounted in 46.2% (18/39) cases in the TVM group and uterine prolapse alone in 56.8% (21/37) cases in the RSC group (Table 1).

**Table 1:** Comparison of general clinical data

	TVM group (n = 39)	RSC group (n = 37)	p-value
Age (years)	72.0 (47–87)	73.0 (56–86)	0.963
BMI (kg/m^2^)	24.7 (18.5–32.7)	24.5 (17.9–32.0)	0.868
POP-Q stage			< 0.005
Stage I	0 (0%)	0 (0%)	
Stage II	9 (23.1%)	21 (56.7%)	
Stage III	24 (61.5%)	11 (29.7%)	
Stage IV	6 (15.4%)	5 (13.5%)	
Type of POP			< 0.05
Cystocele	18 (46.2%)	2 (5.4%)	
Uterine prolapse	0 (0%)	21 (56.8%)	
Cystocele + uterine prolapse	19 (48.7%)	13 (35.1%)	
Cystocele + rectocele	1 (2.6%)	1 (2.7%)	
Cystocele + uterine prolapse + rectocele	1 (2.6%)	0 (0%)	

The median operative time was slightly longer in the RSC group (TVM vs. RSC = 78.0 vs. 111.0 min), but the difference was not statistically significant (p = 0.063) (Table 2). The amount of blood loss was 20.0 ml vs. 5.0 ml (p < 0.005) and the postoperative hospital stay was 4.0 days vs. 3.0 days (p < 0.005), (Table 2).

**Table 2:** Comparison of perioperative parameters

	TVM group (n = 39)	RSC group (n = 37)	p-value
Operation time (min)	78.0 (47–209)	111.0 (84–179)	0.063
Blood loss (ml)	20.0 (5–490)	5.0 (5–80)	< 0.005
Postoperative hospital stay (day)	4.0 (4–5)	3.0 (2–5)	< 0.005

The incidence of postoperative complications was no difference between TVM and RSC group [17.9% (7/39) vs. 16.2% (6/37) (p = 1.000)] (Table 3), of which the recurrence rate of POP was 15.4% (6/39) vs. 13.5% (5/37) (p = 1.000). One case occurred mesh exposure in the TVM group and one case of umbilical hernia in RSC group (Table 3).

**Table 3:** Comparison of postoperative complication (cases number, %)

	TVM group (n = 39)	RSC group (n = 37)	p-value
Total complications	7 (17.9%)	6 (16.2%)	1.000
Postoperative recurrence	6 (15.4%)	5 (13.5%)	1.000
Uterine prolapse	2 (5.1%)	0 (0%)	
Cystocele	0 (0%)	4 (10.8%)	
Rectocele	4 (10.3%)	1 (2.7%)	
Mesh exposure	1 (2.6%)	0 (0%)	
Umbilical hernia	–	1 (2.7%)	

The percentage of patients with preoperative SUI was 15.4% (6/39) vs. 10.8% (4/37) (p = 0.737) in the TVM and RSC groups (Table 4). The improved rate of postoperative SUI was 10.3% (4/39) vs. 8.1% (3/37). Two out of 39 (5.1%) in TVM and 1 out of 37 (2.7%) in RSC were persistent. The incidence rate of postoperative de novo SUI within 12 months after surgery was 25.6% (10/39) vs. 5.4% (2/37) (p = 0.025) in the TVM and RSC groups (Table 4). The incidence rate of de novo SUI within 3 months after surgery was 23.1% (9/39) vs. 5.4% (2/37) in the TVM and RSC groups (p < 0.05) (Table 4).

**Table 4:** Comparison of stress urinary incontinence (cases number, %)

	TVM group (n = 39)	RSC group (n = 37)	p-value
Preoperative SUI	6 (15.4%)	4 (10.8%)	0.737
Improved	4 (10.3%)	3 (8.1%)	
Persistent	2 (5.1%)	1 (2.7%)	
De novo SUI	10 (25.6%)	2 (5.4%)	0.025
Within 1 month	8 (20.5%)	1 (2.7%)	
1–3 month	1 (2.6%)	1 (2.7%)	
After 3 month	1 (2.6%)	0 (0%)	

## Discussion

POP incidence has been increased with advancing age [2], so the prevalence rate of POP is predicted to increase in the aging society in Japan. Not only age, childbearing history, and obesity are reported as risks for POP, the prevalence rate of POP was reported 3–50% in the previous study [1]. This study showed that the surgical time was slightly longer in RSC, but the amount of blood loss and hospital stay was the same compared with TVM. According to a meta-analysis of LSC vs. RSC, RSC was associated with significantly lower blood loss, more operative time, and no differences were observed in postoperative complications and recurrence rate compared with LSC [10].

Wei and Maher et al. reported that the comparison of surgical outcomes between TVM and LSC resulted in longer operative time, less blood loss, and shorter hospital stay [11, 12]. Patients who underwent LSC recovered more quickly and had less recurrence than patients who underwent TVM, although it requires more time for surgery [11]. To compare with the previous study, only blood loss was less in our study, no significant differences were observed in operation time and postoperative hospital stay [11].

The advantages of robotic surgery include the ability to obtain a 3D magnified field of view and the wide range of motion of the EndoWrists. These can allow for less invasive and more safe surgery than laparoscopic surgery. For example, the anterior longitudinal ligament, where the mesh is sutured and fixed in sacrocolpopexy, is difficult to see in laparoscopic surgery, but easily visible in robotic surgery. Therefore, RSC has the potential to be less invasive and to reduce operative time, blood loss, and hospital stay.

The postoperative recurrence rate of TVM and LSC reported by Wei was 8.6% vs. 5.0% (p = 0.064) no significant differences were observed, and our study of TVM vs. RSC showed similar result [11]. In this study, there was no difference in improved and persistent preoperative SUI between TVM and RSC groups. But the incidence rate of de novo SUI was 25.6% for TVM and 5.4% for RSC. We could not find a study that compares the occurrence rate of SUI by TVM and RSC. The incidence of de novo SUI in TVM and LSC varies widely in the literature, with Wei et al. reported 12.0% vs. 12.0% [11], Chen et al. reported 9.5% vs. 5.0% [13], and Sato et al. reported 32.0% vs. 29.7% [14].

De novo SUI after POP surgery is difficult to predict its occurrence preoperatively. Although preoperative urodynamic studies and assessment of SUI appearing on stress testing with corrected organ prolapse are useful for evaluating de novo SUI, they are not sufficient to predict all SUI [15]. Excessive tension on the bladder neck during mesh fixation, which alters the morphology of the urethra, has been suggested as a risk factor for the development of de novo SUI [7, 16]. However, none of the reports specifically describes appropriate tension.

Our result and the results of LSC from the literature suggested that RSC reduces the incidence of de novo SUI more than TVM and LSC.

De novo SUI has occurred within 3 months postoperatively in our study. In our hospital, patients are followed up at 1 month, 3 months, and 6 months postoperatively. To collect more accurate data on de novo SUI and to intervene, it would be useful to evaluate lower urinary tract symptoms preoperatively and at 1 month and 3 months postoperatively.

However, our trial has several limitations. First, this analysis is a retrospective study. We only collected data on the medical charts of our hospital. Some patients may have not told their doctor their symptoms or may have visited another hospital.

Second, in comparison of clinical data, there were difference between TVM and RSC groups in terms of POP-Q staging and type of POP, it may have affected the result. We assessed that it depends on type of surgery deal in each department, TVM of urologist and RSC of gynecologist, and did not affect greatly on comparison of surgical outcomes in this study. Other clinical data and operative data was similar between the two groups. Third, we did not do a urodynamics study and did not use an objective assessment index for SUI such as OABSS (overactive bladder symptom score) and the ICIQ-SF (International Consultation on In-continence Questionnaire-Short Form) for diagnosis of SUI. It leads that the method of diagnosis of SUI may differ depending on the doctor.

## Conclusion

RSC is more useful less invasive surgical method than TVM for patient suffering from pelvic organ prolapse in terms of decreasing blood loss and hospital stay. In addition, RSC caused less de novo SUI as postoperative complication of RSC than TVM.

## Data Availability

The datasets used during the current study are not publicly available due to the protection of personal information of the patients, but are available from the corresponding author on reasonable request.

## Abbreviations

POP: Pelvic organ prolapse
QOL: Quality of life
POP-Q: Pelvic organ prolapse quantification
TVM: Transvaginal mesh surgery
LSC: Laparoscopic sacrocolpopexy
RSC: Robot-assisted sacrocolpopexy
SUI: Stress urinary incontinence
OABSS: Overactive bladder symptom score
ICIQ-SF: International Consultation on In-continence Questionnaire-Short Form
